# Molecular analysis and intestinal expression of *SAR1 *genes and proteins in Anderson's disease (Chylomicron retention disease)

**DOI:** 10.1186/1750-1172-6-1

**Published:** 2011-01-14

**Authors:** Amandine Georges, Jessica Bonneau, Dominique Bonnefont-Rousselot, Jacqueline Champigneulle, Jean P Rabès, Marianne Abifadel, Thomas Aparicio, Jean C Guenedet, Eric Bruckert, Catherine Boileau, Alain Morali, Mathilde Varret, Lawrence P Aggerbeck, Marie E Samson-Bouma

**Affiliations:** 1 Service de Médecine Infantile 3 et Génétique Clinique, INSERM U954, Hôpital d'Enfants Brabois, CHU Nancy, Vandoeuvre les Nancy, 54511, France; 2 INSERM U781, Université Paris Descartes, Hôpital Necker Enfants Malades, Paris, 75015, France; 3 UF de Biochimie des Maladies Métaboliques, Service de Biochimie Métabolique, Groupe Hospitalier Pitié-Salpêtrière (AP-HP), and Département de Biologie Expérimentale, Métabolique et Clinique, EA 4466, Faculté des Sciences Pharmaceutiques et Biologiques, Université Paris Descartes, Paris, 75013, France; 4 Laboratoire d'Anatomie et de Cytologie Pathologiques, Hôpital de Brabois, Université Paris 13, Bobigny, 93000, France; 5 Service de Gastroentérologie, Hôpital Avicenne, 125 rue de Stalingrad, Université Paris 13, Bobigny, 93000, France; 6 Service d'Endocrinologie-Métabolisme, Hôpital Pitié Salpêtrière, (AP-HP), Paris, 75013, France; 7 INSERM U747, Université Paris Descartes, Paris, 75006, France; 8 Service de Biochimie et Génétique Moléculaire, CHU A Paré, AP-HP et Faculté de Médecine (PIFO-UVSQ), Boulogne, 92104, France; 9 Service de Microscopie Electronique, Hôpital de Brabois, CHU Nancy, Vandoeuvre les Nancy, 54511, France

## Abstract

**Background:**

Anderson's disease (AD) or chylomicron retention disease (CMRD) is a very rare hereditary lipid malabsorption syndrome. In order to discover novel mutations in the *SAR1B *gene and to evaluate the expression, as compared to healthy subjects, of the Sar1 gene and protein paralogues in the intestine, we investigated three previously undescribed individuals with the disease.

**Methods:**

The *SAR1B, SAR1A *and *PCSK9 *genes were sequenced. The expression of the *SAR1B *and *SAR1A *genes in intestinal biopsies of both normal individuals and patients was measured by RTqPCR. Immunohistochemistry using antibodies to recombinant Sar1 protein was used to evaluate the expression and localization of the Sar1 paralogues in the duodenal biopsies.

**Results:**

Two patients had a novel *SAR1B *mutation (p.Asp48ThrfsX17). The third patient, who had a previously described *SAR1B *mutation (p.Leu28ArgfsX7), also had a p.Leu21dup variant of the *PCSK9 *gene. The expression of the *SAR1B *gene in duodenal biopsies from an AD/CMRD patient was significantly decreased whereas the expression of the *SAR1A *gene was significantly increased, as compared to healthy individuals. The Sar1 proteins were present in decreased amounts in enterocytes in duodenal biopsies from the patients as compared to those from healthy subjects.

**Conclusions:**

Although the proteins encoded by the *SAR1A *and *SAR1B *genes are 90% identical, the increased expression of the *SAR1A *gene in AD/CMRD does not appear to compensate for the lack of the SAR1B protein. The PCSK9 variant, although reported to be associated with low levels of cholesterol, does not appear to exert any additional effect in this patient. The results provide further insight into the tissue-specific nature of AD/CMRD.

## Background

Anderson' disease (AD) (OMIM 246700) or Chylomicron Retention Disease (CMRD) are the terms used to describe a disorder characterized by hypobetalipoproteinemia with selective absence of apoB48 in the post prandial state [1-26]. It is a very rare recessively inherited disease with less than 50 cases having been reported in the literature. Subjects with this disorder exhibit the clinical manifestations initially described by Anderson and her colleagues which consist of a malabsorption syndrome with steatorrhea and failure to thrive [1]. Endoscopy shows a typical white stippling, like hoar frosting, covering the mucosal surface of the small intestine. The enterocytes in intestinal biopsies contain accumulations of large lipid droplets free in the cytoplasm as well as membrane-bound lipoprotein-sized structures [2,8,10-14,17]. Neuro-retinal manifestations are occasionally present in young patients [8,10,11,19,24]. However, neurological signs may develop more frequently later in untreated individuals and consist most frequently of the loss of deep tendon reflexes [8,10,19,24]. When diagnosis and treatment do not occur until adulthood, neurological signs, including areflexia, ataxia and myopathy, may be more severe [4,5,21]. Recently, myolysis was reported in 8 patients with AD [21]. In all the patients reported in the literature, there is an absence of apoB48-containing lipoproteins. ApoB100-containing lipoproteins are present, although frequently in decreased amounts. There are low levels of plasma high density lipoprotein (HDL)-cholesterol, total lipids, cholesterol, phospholipids, carotenoids and lipid soluble vitamins (particularly vitamin E) whereas fasting triglyceride levels are in the low normal range. Plasma apoB100 and apoAI levels are 20-70% of normal. Increased amounts of apoB48, apoAI and apoAIV have been found in enterocytes [5,6,8]. Acanthocytosis is exceptional and there have been no reports of retinitis pigmentosa. A low fat diet supplemented with lipid soluble vitamins (A and E) results in the resumption of normal growth with abatement of the gastrointestinal symptoms.

In several patients (Table as Additional file 1), the molecular basis for the defect in chylomicron secretion has been shown to be a mutation in the *SAR1B (*formerl*y SARA2) *gene which encodes the SAR1B protein [18-24,26]. This protein belongs to the Sar1-ADP-ribosylation factor family of small GTPases and it is involved in the vesicular coat protein complex II (COPII)-dependent transport of proteins from the endoplasmic reticulum to the Golgi apparatus [27-30]. Recent studies of chylomicron assembly have shown that the Sar1/COPII protein complex also is required for fusion of the specific chylomicron transport vesicle, the PCTV (pre-chylomicron transport vesicle), with the Golgi [31-35]. The *SAR1B *gene (OMIM 607690) is located at 5q31.1. It is composed of 8 exons and alternative splicing of exon 2 is predicted to lead to two transcripts (NM_001033503, NM_016103). To date, 15 *SAR1B *gene mutations (frameshifts, missense or deletions, Figure as Additional file 2) have been described in patients with AD or CMRD [18-24]. A second human protein isoform of Sar1, SAR1A, is encoded by the *SAR1A *gene (OMIM 607691) which is located at 10q22.1. *SAR1A *also is composed of 8 exons. As in the case of *SAR1B*, alternative splicing of exon 2 is predicted to lead to two transcripts (NM_001142648, NM_020150). To date, no mutation in the *SAR1A *gene has been described in patients with AD/CMRD.

We describe here three individuals with mutations in the *SAR1B *gene from two previously undescribed families. Two children in the first family carry a novel mutation. In the second family, one child carries a previously described mutation in the *SAR1B *gene and, unexpectedly, a polymorphism in the *PCSK9 *gene (Proprotein convertase subtilisin/kexin type 9). This polymorphism is of interest because heterozygous variations in the *PCSK9 *gene have been found, recently, to be a major additional determinant of circulating levels of LDL-cholesterol in humans [36-39].

Finally, for the first time, we describe the differential expression of the *SAR1A *and *SAR1B *genes and the localization of the Sar1 proteins in the enterocytes of intestinal biopsies from Anderson's disease patients as compared to healthy individuals.

## Patients and Methods

### Patients

**Patient AD1**, a boy born in 1998, was the first of 3 children of Turkish consanguineous first degree parents. The plasma total cholesterol levels of the father (2.4 g/L) and the mother (2.6 g/L) and their LDL cholesterol levels (2.0 and 1.55 g/L, respectively) were in the normal range for the laboratory. The patient was admitted to the university hospital at 10 months of age with chronic diarrhea (from the age of 3 months) as the main clinical symptom along with moderate growth retardation (-1SD in weight) and a distended abdomen. The neurological examination was normal.

**Patient AD2**, a girl born in 2003, is AD1's sister. She was first admitted to the hospital at one month of age for moderate growth retardation (-1SD in weight), sub-acute diarrhea and a distended abdomen.

**Patient AD3**, a girl born in 1990, was the last of 8 children from unrelated Moroccan parents. The mother's cholesterol level was normal. The father is no longer a participant in the family and his lipid levels are not available. Diarrhea, which began at 3 months of age, was the main clinical symptom. At 8 months of age, the patient was hospitalized for growth retardation (-2 SD in weight and -2 SD in height) and a distended abdomen.

Blood samples and intestinal biopsies were collected from the patients and normal control subjects, using the procedures and the experimental methods approved by INSERM (RBM 0256) and by a bioethics committee (Comité Consultatif de Protection des Personnes dans la Recherche Biomédicale de Paris Bichat-Claude Bernard, Paris, France, CCPPRB Bichat-C. Bernard-2003/05). Informed, written consent was obtained from all participants or from their legal guardians.

### Intestinal biopsies

For diagnostic purposes, duodenal biopsies were obtained, after a 12-15 hour fast, from the patients and from 7 healthy subjects (2 children, 4 and 8 years old, respectively, 1 young adult, 23 years old and 4 older adults, 54 to 64 years old) undergoing endoscopy for causes unrelated to malabsorption and who were following their normal diet. Duodenal biopsies either were fixed in buffered formalin and further embedded in paraffin for standard histology and immunohistochemistry or were directly frozen in liquid nitrogen for oil red O staining of the lipids and for the analysis of intestinal *SAR1A *and *SAR1B *gene and protein expression. Electron microscopy of the biopsies was performed as previously described [14].

### Lipid and lipoprotein analysis

Venous blood samples were drawn after a 12-hour fast (T0) and then 90 min. (T90) and 180 min. (T180) after a standard lipid load. The lipid load was a 620 kcal-meal that was given at T0 immediately after the drawing of the fasting blood samples. It contained about 66% lipids, with monounsaturated fatty acids present in a higher proportion than the saturated fatty acids. Typically, it was composed of 200 mL of unskimmed milk with sweetener, 50 g of bread, 10 g of butter and 100 g of cream (30% fat) with sweetener. Total cholesterol (TC) and triglyceride concentrations were determined by automated enzymatic methods (Konelab, Thermoclinical Labsystems, Cergy Pontoise, France, and Biomerieux, Marcy L'Etoile, France, respectively) [40,41]. HDL-cholesterol (HDL-C) was determined by a direct method according to Egloff et al. [42]. LDL-cholesterol (LDL-C) was calculated using Friedewald's equation [43]. ApoAI and apoB were measured by immunonephelometry using anti-apoAI and anti-apoB antisera (Dade-Behring) and a BN II nephelometer analyzer from Behring (Dade Behring, Marburg GmbH, Germany) [44]. Total lipoproteins were isolated by ultracentrifugation (d < 1.21 g/mL for 4 hours at 90,000 rpm in a NVT 90 Beckman rotor) and analyzed by SDS-polyacrylamide gel electrophoresis. Western blotting was performed with three monoclonal antibodies (generous gift of Y. Marcel) to detect the presence of apoB48, apoB100 and possible truncated forms of apoB [45,46]: the antibodies are specific for 1) the N-terminal side of apoB100 and apo B48 (antibody 1D1 recognizes epitopes located between amino acids 474 and 539 of the protein), 2) for the C-terminal side of apoB100 (antibody L3 recognizes the epitopes close to amino acid 4355) and 3) for the central part of apoB (antibody L9 is specific for amino acids located between residues 2835 and 2922 present in both apoB48 and apoB100).

### Gene sequencing of SAR1A, SAR1B, PCSK9 and exon 7 of the LDLR

Genomic DNA was isolated from whole blood containing EDTA with a PSS Magtration 8 Lx machine using a magnetic marble technique, according to the manufacturer's instructions. The 8 exons of *SAR1A*, the 8 exons of *SAR1B*, the 12 exons of *PCSK9 *and exon 7 of the *LDLR *genes and their flanking intronic sequences, were amplified by the polymerase chain reaction (primer sequences and PCR conditions are available on request). These genes were tested because of their known roles in the secretory pathway and/or their involvement in hypocholesterolemic syndromes. Exon 7 of the *LDLR *gene encodes the EGF A repeat domain of the LDL receptor which is essential for PCSK9 binding. Bidirectional fluorescent sequencing of purified PCR products (ExoSAP-IT, GE Healthcare) was carried out with the Big Dye^® ^Terminator v1.1 Cycle Sequencing kit on an ABI-PRISM^® ^3130XL genetic analyzer (Applied Biosystems, Applera France SA). Electrophoretograms were analyzed using Gensearch DNA sequence analysis software (Phenosystems SA., Belgium).

### Immunohistochemical studies of the intestinal biopsies

The localization of Sar1 proteins in duodenal biopsies from the 3 AD patients and from 3 of the healthy subjects was assessed by immunostaining with Nova Red (VECTOR) using a chicken polyclonal antibody against recombinant human SAR1A protein (ABCAM 14270, dilution 1:100) and anti-chicken horseradish peroxidase-conjugated antibody (ABCAM 6877, dilution 1:2000).

### Expression of the SAR1A and SAR1B genes in the intestine

For the analysis of gene expression, intestinal biopsies were frozen immediately in liquid nitrogen and then stored in liquid nitrogen or at -80°C. Total RNA was extracted from the frozen intestinal biopsies using the RNeasy Mini kit (Qiagen) and treated according to the manufacturer's recommendations. The quantity and quality of the RNA were assessed using a Nanodrop ND-1000 spectrophotometer (Thermo Fisher Scientific Inc.). The integrity of the RNA was evaluated by agarose gel electrophoresis. Reverse transcription was performed using the Superscript II Reverse Transcriptase protocol for random hexamer-mediated cDNA synthesis (Invitrogen).

Real time quantitative PCR was performed with the ABI Prism model 7300 Sequence Detection System (Applied Biosystems), using the Taqman MGB specific probes for *SAR1A *(Hs00833068_s1), for *SAR1B *(Hs01011583_m1) (Applied Biosystems), and Absolute blue QPCR ROX mix (Abgene). The *GAPDH *(Hs99999905_m1) and *POLR2A *(Hs00172187_m1) genes were used as housekeeping control genes after validation (Applied Biosystems). The assays amplified single, gene-specific amplicons of the correct sizes (without non-specific products) and with uniform PCR efficiencies. All probes were validated in triplicate by the use of serial cDNA dilutions. Target and reference amplifications exhibited 100 ± 5% efficiency (R^2^>99% for the standard curve). The range of the Ct values was 10-35. The standard deviation for the Ct values of each sample was less than 0.3. Each sample was analyzed in triplicate and then differences in the expression of each gene among the samples were quantified using the (ΔΔCt) approach using both endogenous controls *GAPDH *and *POLR2A *(both gave similar results) [47]. The difference in expression between the AD sample and the sample from each healthy subject was evaluated by Student's t test.

## Results

The diagnosis of Anderson's disease in the three patients was made on the basis of the observations described below which, collectively, are pathognomonic for the disease. Celiac disease, cystic fibrosis and cow's milk allergy were all ruled out.

### Biological results and intestinal structure and ultrastructure

**Figure 1:**
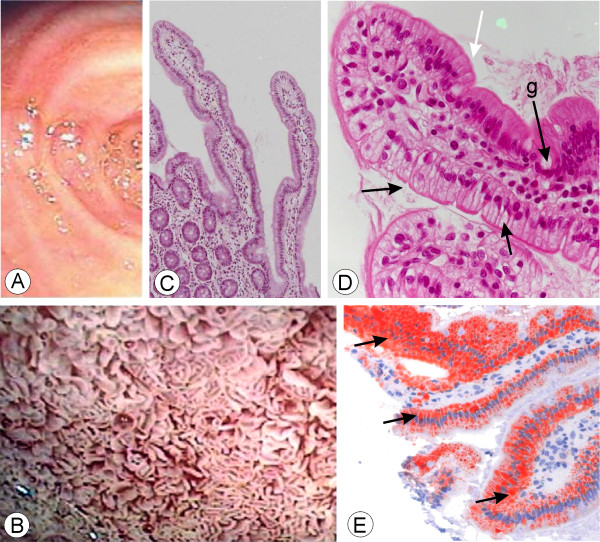
**Intestinal endoscopy after a 12-hour fast**. In contrast to what is observed in a normal subject (a), video-endoscopy of the duodenum (D3) of patient AD2 (b), shows the typical « white hoary frosting » on the small intestinal mucosa. In contrast with a normal subject (c), light microscopy of the duodenal biopsy from AD2 (d) shows the typical vacuolated enterocytes (black arrows) that stain positively with oil red O (e, black arrows). Note the typical heterogeneous aspect of the *villi *either fat loaded (black arrows) or without lipid droplets (white arrows). Goblet cells are normal (d, arrow g). (c ×100; d ×400; e ×200)

**Figure 2:**
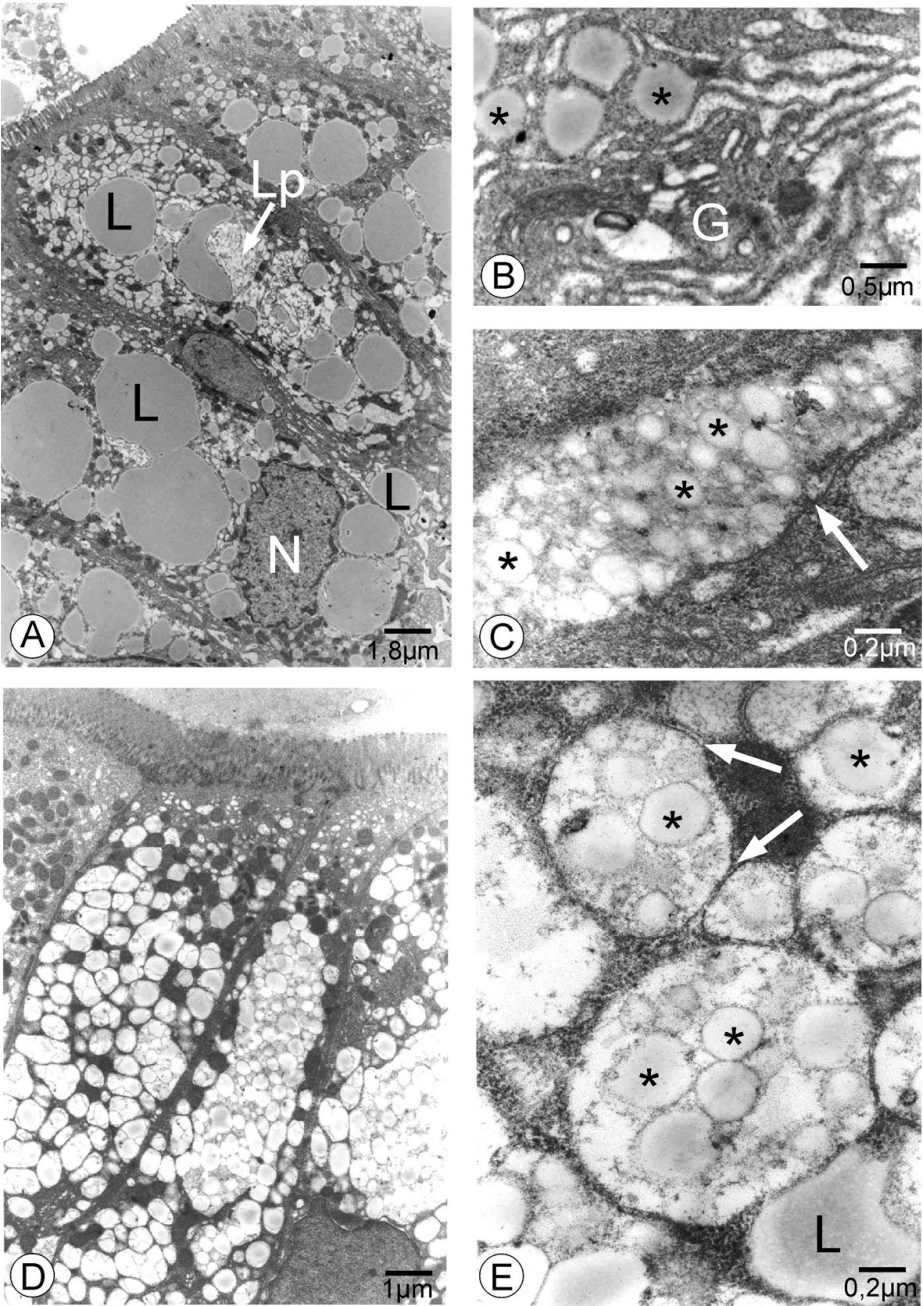
**Electron microscopy of duodenal biopsies of patients with AD**. As shown for AD3 (a, b, c) and AD2 (d, e), two types of particles are apparent in the enterocytes in these patients (a,d): large lipid droplets, free in the cytoplasm (L), and smaller, lipoprotein-sized like particles (Lp), surrounded by a membrane. A higher magnification shows in (b) some individual lipoprotein-sized particles surrounded by a membrane (*) near a Golgi apparatus (G) which appears distended but devoid of particles and in (c,e) numerous lipoprotein-sized particles accumulated in a membrane bound compartment (membrane, white arrow). The intercellular spaces are empty. The cell nucleus is labelled N.

**In patient AD1**, the total vitamin E level as well as the ratio of vitamin E to [cholesterol + triglyceride] were low. The vitamin A level was normal. The plasma total cholesterol, LDL-cholesterol, HDL-cholesterol, and apoAI values were low whereas apoB levels were low normal and triglyceride levels were normal (Table 1). There were a few acanthocytes (<1%). The values for ASAT and CK were slightly increased (Table 1) and the CPK-MB levels were normal. After a 12 - hour fast, intestinal video-endoscopy showed the typical « white hoary frosting » on the mucosa and duodenal biopsies exhibited fat loaded enterocytes typical of those seen in Anderson's disease (shown in Figure 1b for AD2) as compared to normal individuals (Figure 1a). The intestinal biopsy exhibited normal *villi. *Enterocytes from AD patients were overloaded with fat droplets in a heterogeneous manner (enterocytes stained positively with oil red O mainly on one side of the villus) (compare Figure 1c, from a normal individual, with Figure 1d, e). Ultrastructural examination of the biopsies showed that the enterocytes contained an accumulation of numerous free lipid droplets as well as membrane bound lipoprotein-sized structures that accumulated in the enterocytes (similarly as in Figure 2 for AD2 and AD3). At 11 months of age, **v**itamin E supplementation (a-tocopherol 100 mg/kg/d) and a 60% Medium Chain Triglycerides (MCT) formula was initiated. After a few weeks, the diarrhea improved and the steatorrhea ceased. At 4 years of age, exocrine pancreatic insufficiency (EPI) was suspected because of a recurrent lipid malabsorption syndrome. Between 4 and 10 years of age, several slightly decreased fecal elastase levels (70 to 200 μg/g) were noted and steatorrhea was documented on several occasions (maximum 7.4 g/d at 8 years of age).

**Table 1:** Clinical and biological data of Patients.

Patients	AD1	AD2	AD3
Parental consanguinity	yes		no
Age of onset of symptoms	3 months	1 month	3 months
Growth retardation			
Weight	-1 SD	-1 SD	- 2 SD
Height	Normal	Normal	- 2 SD
Digestive symptoms	diarrhea, abdominal distension		
Deep tendon reflexes	+	+	+
Hematological (Acanthocytes)	< 1%	Normal	5%
Steatorrhea (g/24 hr)	2.2	4.9	2
Cholesterol (N: 1.50 - 2.80)	1.01	1.05	0.89
LDL-cholesterol (N: 0.80 - 1.50)	0.57	0.49	0.38
(g/L) HDL-cholesterol (N >0.45)	0.22	0.32	0.27
Triglycerides (N: 0.50 - 1.70)	1.10	1.20	1.20
ApoA-1 (N: 1 - 2.15)	0.70	0.90	0.50
ApoB (N: 0.55 - 1.40)	0.56	0.43	0.82
Vitamin E (N: 8 - 17 mg/L)	1.8	2.8	1
Vitamin E/Lipid (N >1.9 mg/g)	0.9	1.3	0.5
Vitamin A (N: 240 - 900 mg/L)	682	107	254
ASAT (x ULN)	1.5	1.6	1
ALAT (x ULN)	1	1	1
CK (x ULN)	1.7	1.4	1.7

ULN: Upper limit of normal; SD: Standard Deviation; + present in decreasing amounts; ASAT: Aspartate Amino Transferase; ALAT: Alanine Amino Transferase; CK: Creatine kinase

Currently, despite a normal fat diet, the patient (now 11 1/2 years old) is doing well and, surprisingly, no diarrhea has been reported. Following vitamin E (α-tocopherol 50 mg/kg/d) and exogenous pancreatic enzyme supplementation, the plasma vitamin E and fecal elastase levels have become normal and steatorrhea is absent. The total plasma cholesterol (1 g/L), HDL-cholesterol (0.11 g/L) and LDL-cholesterol (0.76 g/L) values, however, remain low. The plasma CK levels are increased (2.6 fold greater than the upper limit of normal) but the CK-MB levels are normal. There are no neuro-ophtalmic, hepatic or cardiac abnormalities.

**In patient AD2**, the plasma concentration of fat-soluble vitamin A and the ratio of vitamin E to total lipids were decreased (Table 1). The plasma total cholesterol, LDL-cholesterol, HDL-cholesterol, apoAI, and apoB levels were low whereas the triglyceride level was normal (Table 1). The values for ASAT and CK were increased whereas CK-MB levels were normal (Table 1). Steatorrhea was present and fecal elastase levels were low (47 μg/g) when the patient was evaluated initially. Exogenous enzyme supplementation did not modify pancreatic function. The video-endoscopy of the intestine and the microscopic appearance of the intestinal biopsy (staining with oil red and electron microscopy), shown in Figures 1 and 2 along with images for AD3, were similar to that of patient AD1. At 2 months of age, the patient was given liposoluble vitamins and MCT supplementation as for AD1.

Currently, the patient (now 7 years old), like her brother, consumes a normal fat diet supplemented with vitamins D, E (α-tocopherol 30 mg/kg/d) and K as well as with exogenous pancreatic enzymes. She is a healthy young girl without digestive symptoms. The values for liposoluble vitamins and fecal elastase are in the normal range and steatorrhea is absent. The plasma total cholesterol, HDL- and LDL-cholesterol remain low (1.2, 0.18 and 0.86 g/L, respectively). The plasma CK and ASAT levels are increased (1.2 fold and 1.4 fold greater than the upper limit of normal, respectively) whereas the CK-MB levels are normal. Similarly to her brother, there are no neuro-ophtalmic, hepatic or cardiac abnormalities.

**In patient AD3**, there was a very low ratio of vitamin E to [cholesterol + triglyceride] whereas the vitamin A level was normal (Table 1). The plasma total cholesterol, LDL-cholesterol, HDL-cholesterol and apoAI levels were very low, whereas triglycerides and apoB levels were normal (Table 1). Fecal elastase level was normal. Anemia (normochromic, normocytic and then microcytic) with decreased serum iron and decreased saturation was present. A few acanthocytes were observed (5%). The video-endoscopy of the intestine and the microscopic appearance of the intestinal biopsy (staining with oil red and electron microscopy) resembled that of patient AD2 (Figures 1 and 2). A low fat diet as well as liposoluble vitamin supplementation (a-tocopherol 60 mg/kg/j) was initiated at 9 months of age.

Currently, the patient (now 19 ½ years old) follows a self-imposed low fat diet with vitamin D and E (oral tocofersolan 300 mg/d) supplementation but without strict adherence. The growth curve remains below the normal range (-1.5 SD) which, however, must be interpreted in the context of a short familial and ethnic height (BMI 30^th ^percentile). The digestive symptoms now have disappeared. The deep tendon reflexes are present and there are no muscular symptoms. There is no steatorrhea and the fecal elastase level is normal. The plasma total cholesterol, HDL-cholesterol and LDL-cholesterol levels remain low (0.68, 0.14, 0.39 g/L respectively). The ratio of vitamin E to total lipids and the vitamin D level (55 μg/L) are now normal. The electromyogram shows neither neurogenic nor myogenic abnormalities. Nerve conduction velocity is normal. The CK levels are slightly increased (2-fold higher than the upper limit of normal) although CK-MB and transaminase levels are normal. There is no hepatic steatosis (normal abdominal ultrasound), no cardiomyopathy (normal cardiac ultrasound) and no ophthalmic abnormality.

Either fasting or following a fat load, the patients exhibited lipid and apolipoprotein levels typical of those reported for other individuals with Anderson's disease [6]. In patients AD1 and AD3, there was no increase in triglyceride levels at 90 and 180 minutes after the fat meal as compared to the fasting value (Table 2). Typically, in normolipidemic subjects, although a fat load produces no significant change in total cholesterol concentration, the triglycerides increase after a fat meal [48]. No chylomicrons were observed following the lipid-rich meal in patients AD1 and AD3, whereas in healthy normolipidemic subjects chylomicrons are easily demonstrable 90 and 180 minutes after the fat load. SDS polyacrylamide gel electrophoresis of lipoproteins from the patients following the fat load showed that apoB100 was present whereas apoB48 was undetectable at all the times studied. No truncated form of apoB was detected at any time.

**Table 2:** Plasma lipid, apolipoprotein, and lipoprotein analysis in two AD subjects after a fat load*.

Patients	AD1			AD3			Fasting normal range	After a fat load, T90-T180
	**T0**	**T90**	**T180**	**T0**	**T90**	**T180**		
**Total cholesterol**	1.06	1.03	1.09	0.90	0.91	0.94	1.20-2.20	No change
**LDL-cholesterol**	0.73	0.70	0.76	0.56	0.56	0.62	0.40-1.10	
**HDL-cholesterol**	0.21	0.22	0.21	0.19	0.20	0.19	0.40-0.65	
**Triglycerides**	0.58	0.57	0.58	0.74	0.74	0.66	0.35-1.30	1.68-2.04
**ApoA-I**	0.71	0.68	0.67	0.65	0.63	0.59	1.30-1.80	
**ApoB**	0.68	0.65	0.65	0.57	0.58	0.53	0.50-1.00	
**Chylomicrons in serum**	Absent	Absent	Absent	Absent	Absent	Absent	Absent	Present
**Presence of apoB48 in isolated lipoproteins**	No	No	No	No	No	No	No	Yes
**Presence of apoB100 in isolated lipoproteins**	Yes	Yes	Yes	Yes	Yes	Yes	Yes	Yes

*Values are given in g/L.

As far as can be determined from the literature, the acquisition of tolerance to fat in the diet is limited to only a few cases (the two reported here and [[11,12], and [22]]). In all the other instances reported, the patients' gastrointestinal symptoms recur when fat is reintroduced into the diet. Charcosset and colleagues [19] have proposed that network(s) of modifier genes are involved in the mechanism(s) that determine(s) the phenotype of the disease in a given individual. Whether this could be a mechanism that is involved in the acquisition of tolerance to fat is unknown at present.

### Mutations in the SAR1B and PCSK9 genes

**Figure 3:**
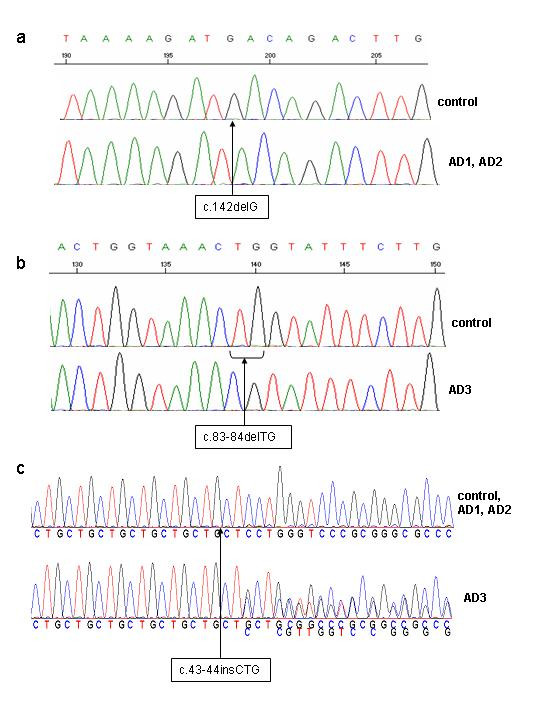
**Mutations in the SAR1B and PCSK9 genes**. Electropherograms show (a) the c.142delG mutation in the *SAR1B *gene of patients AD1 and AD2, (b) the c.83_84delTG mutation in the *SAR1B *gene of patient AD3 and (c) the c.61_63dupCTG polymorphism in the *PCSK9 *gene of patient AD3.

Sequencing of all the exons of the *SAR1A *and *SAR1B *genes, including the intron-exon boundaries revealed that the two siblings, **patients AD1 and AD2**, had a mutation in exon 4 (formerly exon 3) of the *SAR1B *gene. There is a homozygous deletion at position 142 (c.142delG) of the *SAR1B *gene (Figure 3a). This deletion results in the replacement of the aspartic acid at position 48 by a threonine and also gives rise to a frameshift resulting in a premature stop codon 17 amino acids further on (p.Asp48ThrfsX17). This is a novel mutation in the *SAR1B *gene and gives rise to a truncated protein that has only 32% of the length of the normal protein and comprises only 24% of the normal sequence.

**Patient AD3 **has a homozygous two base pair deletion (c.83_84delTG) in exon 4 (formerly exon 3) of the *SAR1B *gene (Figure 3b). This deletion substitutes an arginine for the leucine normally present at position 28. A frameshift, resulting in a premature stop codon (p.Leu28ArgfsX7), produces a truncated protein that has only 17% of the length of the normal protein and 14% of the normal sequence. This mutation has been reported previously in patients of European and Moroccan origin [18,23] (Table as Additional file 1). Because of their different ethnic origins, it is very unlikely that the mutational event reported here and in Cefalu et al. in patients from North Africa are the same as the one described by Jones et al..

The *PCSK9 *gene was also sequenced in these patients because it has been found to be a determinant in the levels of circulating LDL-cholesterol. In patient AD3, there was also a hypocholesterolemic variant in the *PCSK9 *gene. AD3 is a heterozygous carrier of a 3-base duplication (c.61_63dupCTG formerly designated c.43_44insCTG) in the first exon of *PCSK9 *(Figure 3c). The predicted amino acid change is a leucine duplication (p.Leu21dup formerly designated p.L15_16insL) in the leucine stretch of the PCSK9 signal peptide which leads to 10 leucine repeats (allele denoted L10) instead of the nine leucine repeats (L9) in the normal allele. This *PCSK9 *hypocholesterolemic variant has been reported previously [49]. There was no mutation in the 12 exons or intron-exon boundaries of PCSK9 in patients AD1 and AD2.

Finally no mutation in the *SAR1A *gene or in exon 7 of the *LDLR *gene was detected in any of the three patients.

### Distribution of the Sar1 proteins in duodenal biopsies from normal individuals and AD patients

**Figure 4:**
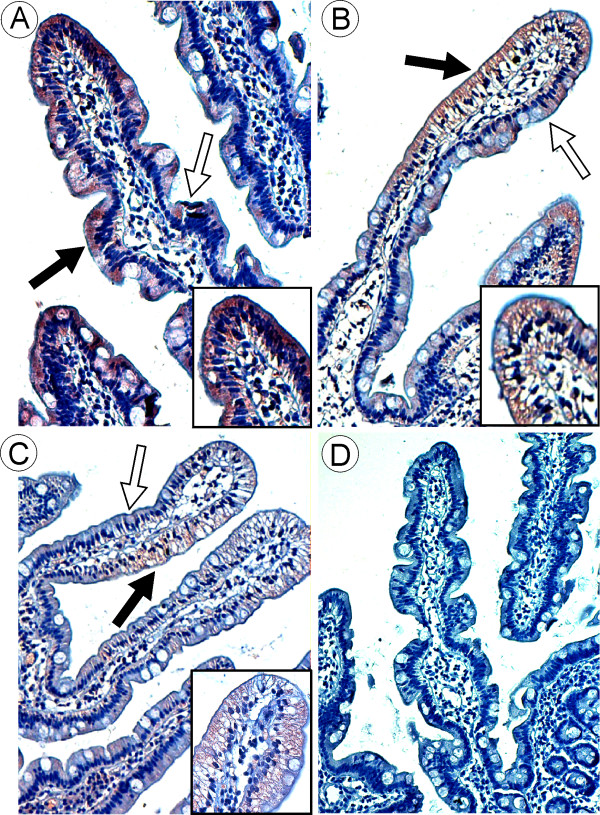
**Distribution of Sar1 proteins in duodenal biopsies from healthy individuals and AD patients**. Immunostaining for Sar1 proteins in normal control subjects (a) was detected mainly in the *villus *tip (black arrows) in the apical part of the enterocytes. The *villi *exhibit a heterogeneous aspect: immunolabelling occurs predominantly on one side (black arrows) and is largely absent on the other side (empty arrows). In patients with AD, AD2 (b) and AD3 (c), the immunolabelling for Sar1 was present in the same area of the *villi *but was clearly decreased in intensity as compared to normal individuals. The immunostaining was detected around the large lipid droplets which distended the cytoplasm of the enterocytes. The same heterogeneous aspect observed in the normal individuals was also seen in the AD subject with staining appearing as slightly positive on one side (black arrows) and negative on the other side (empty arrows). No staining was seen when the primary antibody was omitted (d). (Fig. a ×20; b, c, d, × 10; insert × 40).

In the healthy subjects, the Sar1 proteins were detected readily in the duodenal epithelium. The immunostaining, present mainly in the upper two-thirds of the *villi*, was localized essentially within the apical area of the cytoplasm of the enterocytes underlying the brush border (Figure 4a). The distribution of the Sar1 proteins was heterogeneous along the *villi *with staining present predominantly on one side of the *villus*. All the control reactions were negative and no immunostaining was detected in the brush border or in the goblet cells (Figure 4d).

In the 3 patients with Anderson's disease, the enterocytes were distended by large accumulated lipid droplets. As in the case of healthy individuals, the immunostaining for the Sar1 proteins was heterogeneously distributed along the *villi. *However, the staining was decreased as compared to the healthy subjects and was restricted to the area surrounding the lipid droplets (Figure 4b, c). The Sar1 isoforms differ by only 20 amino acid residues, which occur throughout the entire sequence of the protein, and the polyclonal antibodies used do not discriminate between the two human protein isoforms, Sar1a and Sar1b. Therefore, we decided to analyse the expression of the *SAR1A *and *SAR1B *genes in the intestinal biopsies.

### Expression of the SAR1A and SAR1B genes in duodenal biopsies

**Figure 5:**
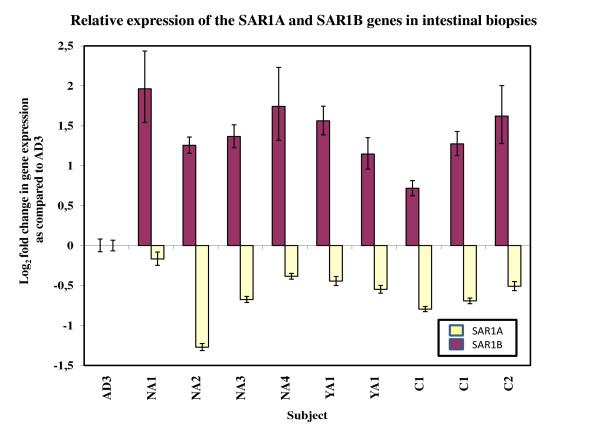
**The expression of the *SAR1A *and *SAR1B *genes in intestinal biopsies of healthy individuals and an Anderson's disease patient**. The graph shows the log base 2 fold change (increase or decrease), as measured by RTQPCR, in the expression of the *SAR1A *and *SAR1B *genes in 4 healthy normal adults (NA1, NA2 NA3 and NA4), one healthy young adult (YA1) and two healthy children (C1 and C2) normalized to an Anderson's disease patient (AD3). Gene expression was measured in biopsies obtained on two separate occasions from the young adult (YA1) and from one of the children (C1). The standard deviation of the measurements are shown and, except for the value for *SAR1A *for NA1, all the differences are significant (p < 0.01) as compared to the patient.

Real time quantitative PCR (Figure 5) showed that the expression of *SAR1B *was significantly (p < 0.01) decreased (between 1.4 and 3.5 fold) in the duodenal biopsies of the Anderson's disease patient AD3, as compared to biopsies of 4 healthy adults (aged 54 to 64 years old), a young adult (23 years old) obtained on two separate occasions and 2 children (4 and 8 years old) one of which was obtained on two separate occasions. In contrast, the level of expression of *SAR1A *was significantly (p < 0.01) increased (between 1.4 and 2.7 fold) in the biopsies of the Anderson's disease patient as compared to biopsies of the same healthy individuals, except for individual NA1 (Figure 5). Biopsies that were taken on two separate occasions from the young adult, YA1, and one of the children, C1, gave similar results. The UniGene EST profile database which provides estimates of mRNA abundances indicates that *SAR1B *is expressed between 2.5 and 3 times the level of *SAR1A *in the intestine. Our results show that, in AD/CMRD, the level of *SAR1B *decreases by almost two thirds whereas *SAR1A *increases by about 1.5 fold. Thus, although *SAR1A *increases, this is not enough to compensate for the decrease in *SAR1B *and the total amount of SAR1 proteins in the intestine is predicted to be less, by about one third, in AD/CMRD patients as compared to normal individuals. These results are, thus, in agreement with the immuno-histochemistry results.

## Discussion

In addition to manifesting the clinical, biochemical and intestinal ultrastructural features typical of AD, including mutations in the *SAR1B *gene, the 3 new patients that we describe here (which have now been followed for 7-10 years) exhibit some heretofore unseen features.

The expression of *SAR1B *in sites other than the intestine [18,50,51] suggests that other clinical manifestations might occur in AD patients, such as the recently reported muscular and cardiac manifestations [21]. As in several other cases of AD/CMRD [1-3,7,10,12-14,21,23], AD3 manifests anemia and decreased levels of serum iron. Although the mechanism is not entirely clear, these observations are of interest in light of a report of the preferential expression of *SAR1B *during erythropoiesis [52] and considering that mutations in SEC23B, with which SAR1 interacts in the COPII secretory coat complex, cause congenital dyserythropoietic anemia type II [53]. Further, two of our patients manifest slight acanthocytosis as do four other cases reported in the literature [5,8,10,13] and two other instances not published. In abetalipoproteinemia (ABL, MIM 200100), acanthocytosis (generally more than 50% [54]) has been proposed to be due to changes in the fluidity of the red blood cell membrane as a consequence of changes in its composition (decreased cholesterol and phospholipids leading to a relative increase in sphingomyelin [55]). The origin of the slight acanthocytosis (always less than 5%) in AD/CMRD is not clear. There is only one investigation of red blood cell lipids in AD/CMRD patients [9] and it showed that although total cholesterol and phospholipids were decreased, there was no variation in the phospholipid composition and the phosphatidylcholine to sphingomyelin ratio was similar to that in normal subjects (but no acanthocytosis was reported in those patients). Other factors may be involved. Normal erythrocytes acquire an acanthocytic form after transfusion into and circulation in ABL patients [56] but incubation of normal erythrocytes with plasma from ABL patients does not result in these modifications [57,58]. This suggests that the mechanism(s) of formation of the acanthocytes in ABL are complex and do not simply result from the passive exchange of lipids between the erythrocyte membrane and the plasma but also may reflect exchange with the vessel wall membranes. There are no acanthocytes in the bone marrow of AD3 [59] which also suggests a peripheral acquisition in AD/CMRD. The decreases in cholesterol and phospholipids in AD/CMRD may be at the limit at which changes in membrane fluidity induce changes in erythrocyte morphology thus making the finding of acanthocytes inconstant and variable in the disorder.

Osteoporosis/osteopenia have been described in several cases of AD [1-3,10,11,14,24] although none of the 3 cases reported here exhibit such abnormalities. In these 3 new patients, except for slightly increased CK levels, there is no neuro-ophtalmic, hepatic or cardiac abnormality. Further, none of the patients have any abnormal clinical or biological signs related to the spleen, kidney or lungs. The retardation in growth, which is milder than previously reported for patients with AD, underscores once more the importance of an early diagnosis which permits the implementation, early in life, of a low fat diet supplemented with liposoluble vitamins.

In both AD1 and AD2, exocrine pancreatic insufficiency (EPI) was suspected because of low fecal elastase levels. Whether and to what extent *SAR1B *gene mutations [60] can affect the levels of fecal elastase is unknown. Even though the low fecal elastase levels in AD1 and AD2 (AD3 had a normal fecal elastase level) could be due to diarrhea-related dilution and there are no other indications of pancreatic dysfunction, investigation of pancreatic function may still be prudent in patients with Anderson's disease or in those with unclear phenotypes.

In addition to a mutation in the *SAR1B *gene, patient AD3 also has a polymorphism in the *PCSK9 *gene (OMIM 607786), which encodes the Proprotein Convertase Subtilisin/Kexin type 9. It is linked to autosomal dominant hypercholesterolemia and is involved in cholesterol homeostasis [36-39] and gain-of-function mutations cause increased degradation of the LDL receptor and result in high levels of LDL cholesterol [61,62]. Some mutations of PCSK9 cause variable degrees of hypocholesterolemia in homozygous or compound heterozygous carriers [63,64]. However, these *PCSK9 *hypocholesterolemias appear to be clinically benign. The *PCSK9 *polymorphism found in patient AD3 was found to be present at a higher frequency in a low LDL-C population than in a normal population and to segregate with the "apoB- negative" Familial Hypobetalipoproteinemia phenotype in 3 families [49]. This polymorphism could be an additional genetic hypocholesterolemic variation that might account for the high variability in lipid levels of some patients with AD. We did not find, in any of the three patients, a mutation in exon 7 of the *LDLR *gene which encodes the EGF A repeat domain of the LDL receptor which is essential for PCSK9 binding [62]. A mutation in this binding site could prevent the degradation of the LDL receptor by PCSK9 and also could lead to hypocholesterolemia due to an increase in LDL catabolism [65]. The variation in the *PCSK9 *gene is the second example of a mutation in the *SAR1B *gene being accompanied by a mutation/variation in another gene. In the cases of CMRD-MSS (Marinesco-Sjogren Syndrome), there is, in addition to a mutation in the *SAR1B *gene, a mutation in the *SIL1 *gene [20]. In these patients, CMRD and MSS are distinct diseases.

Although chylomicron retention disease or Anderson's disease has been shown to be attributable to variations in the *SAR1B *gene, a full understanding of the relationship between genotype and phenotype and the origins of tissue-specific manifestations is lacking.

The *SAR1B *gene mutations in the three AD patients described here are predicted to lead to severely truncated SAR1B proteins that possess only 33 or 63 amino acids, respectively, the first 27 or 47 of which correspond to the amino-terminal sequence of the wild type protein. These truncated proteins lack numerous essential conserved residues that are involved in guanine nucleotide binding, in GTP hydrolysis and for interacting with guanine nucleotide exchange factors (GEF) and GTPase-activating proteins (GAP). We expect that the SAR1B proteins produced are non-functional in these patients. The vast majority of *SAR1B *gene mutations in AD/CMRD affect regions that are involved in nucleotide binding and hydrolysis as well as other functions (see Table and Figure as Additional files 1 and 2). A few others affect primarily regions in proximity to the membrane which would also be suspected to severely alter the function of the protein [12,18,19,22,66].

Sarl plays at least two roles in the secretory pathway. Sar1 is a significant participant in the coat protein complex II (COPII), a multi-protein complex which is responsible for the formation, at the endoplasmic reticulum, of membrane vesicles involved in protein transport (protein transport vesicles, PTVs) to the Golgi compartment [27-30]. Sar1, a small GTPase, associates with the smooth ER membrane via the action of Sec12, a GTP/GDP exchange factor. After having bound GTP, Sar1 binds Sec23-Sec24 (the inner coat) to form a prebudding complex. This complex, via Sec24 and perhaps Sec23 and Sar1, as well, interacts with vesicle cargos. Sec13-Sec31, which form the outer coat, are recruited by the Sar1-Sec23-Sec24 complex, which is membrane bound. The outer coat stimulates the GTPase-activating protein Sec23 which activates Sar1 GTP hydrolysis allowing depolymerisation of the coat and recycling of its components. PTVs transport a wide range of soluble and membrane bound cargo in a COPII dependent manner.

In a novel paradigm, Sar1 plays a role in an intestinal chylomicron-specific transport mechanism that employs a pre-chylomicron transport vesicle (PCTV). However, in this mechanism, it is L-FABP that initiates and organizes the PCTV budding machinery [32] which is modified and regulated, in part, by the phosphorylation of a currently unidentified 9 kDa protein [67]. It is of interest in this context that FABP gene disrupted mice exhibit a delayed appearance of dietary triglycerides in the blood [68,69]. PCTVs, similarly to PTVs require ATP. In contrast to PTV**s**, the PCTVs do not require GTP and the vesicle size is markedly larger (200-500 nm for PCTVs as opposed to 60-90 nm for PTVs). Further, the PCTV does not require Sar1 to bud from the ER but rather employs all the COPII proteins, and in particular Sec24C, for docking and fusion with the *cis*-Golgi [31,33,34,70,71]. Prior binding of Sar1 to PCTVs is apparently necessary for the binding of Sec24C [34]. Finally, VAMP7 is the v-SNARE present on PCTVs, whereas Ykt6 and r22b are the v-SNAREs on PTVs [33].

Given the important role of Sar1 in COPII assembly and in the secretory pathway, it is remarkable that AD/CMRD patients have a pathology which is limited primarily to the intestine. Recent studies of cranio-lenticulo-sutural dysplasia CLSD [28,72,73] and congenital dyserythropoietic anemia type II, CDAII, [53] in humans and craniorachischisis in mice [74] provide important insights. The mutation in CLSD affects SEC23A, and the pathological effects are also limited. There are two paralogues of Sec23 and it appears that the second paralogue, SEC23B, can compensate for the loss of activity of SEC23A. Manifestation of the disease occurs, presumably, in tissues where there is not enough SEC23B to compensate for the loss of the other paralogue. However, in CDAII, in which a specific erythropoietic phenotype is observed, SEC23A can not compensate for the paralogue, SEC23B, which is important in the cell cycle of erythroid cells and their cytokinesis [53]. In contrast, in craniorachischisis, Sec24b is affected but also with rather limited effects (although severe). In this case, transport of a specific cargo is strictly dependent on Sec24b indicating that cargo specificity has evolved among the four Sec24 paralogues.

The question arises as to whether in AD/CMRD and for the case of *SAR1*, for which there are also two human paralogues, the disease becomes manifest only in tissues in which there is insufficient amounts of the second paralogue to compensate for the loss of activity of the first paralogue or whether a specific cargo (chylomicrons) is strictly dependent on a specific paralogue(s) of the secretion machinery. Both the *SAR1A *and *SAR1B *genes are expressed in the intestine [18,50,51]. Our results show that the expression of the *SAR1B *gene in the duodenal biopsies from the patient AD3 was markedly decreased as compared to healthy individuals. In contrast, the expression of the normal *SAR1A *gene was significantly increased in AD3. The increased expression of the *SAR1A *gene and, presumably, of its encoded protein, SAR1A, does not appear to be sufficient to compensate for the defect in SAR1B. This is consistent with a Sar1 paralogue-specific and cargo-specific transport of chylomicrons.

In AD/CMRD, Sar1 paralogue- and cargo-specific transport could explain the rather modest and tissue specific manifestations of the disease. If, in enterocytes, the SAR1B paralogue is used specifically in PCTV vesicles, for the transport of chylomicrons, and if the SAR1A paralogue is used in PTVs, for the transport of small soluble and membrane bound proteins, then mutations in *SAR1B *would be expected to affect primarily or only chylomicron transport. Indeed, it has been shown that SAR1B has a weaker affinity for SEC13-SEC31A which could lead to a less constrained structure [73]. This less constrained structure would allow the formation of larger COPII vesicles (which could transport large cargos such as chylomicrons) as compared to those assembled with SAR1A which has a stronger affinity for SEC13-31A [28,75].

It has been proposed, in fact, that mutated SAR1B protein leads to an absence of chylomicronemia in AD because the PCTVs that are produced in the absence of Sar1 do not fuse with the Golgi and are retained in the cytosol [34]. In contrast, in a COPII-GTP-dependent model and by analogy to the disease CLSD in humans, a mutated Sar1 protein might be expected to lead to the accumulation of tubular extensions, devoid of a coat, projecting from distended peripheral ER compartments as a consequence of defective budding [28,72,73]. Electron micrographs of appropriate resolution might be able to distinguish between these two possibilities but, to the present, the heavily lipid-laden nature of the enterocytes obtained from patients with AD, even in the fasted state, has precluded this.

With respect to the liver, the lack of hepatic manifestations in most cases of AD, (hepatic steatosis has been reported in only 4 patients [8,12]) could be explained by differences in intestinal and hepatic trafficking pathways which might also use different COPII paralogues and, in particular, SAR1A for the secretion of apoB100-containing lipoproteins. It has been proposed that a smaller VLDL-specific transport vesicle, the VTV, requires Sar1 (it is not clear as to the isoform involved) to exit from the ER and also involves the ER-to Golgi vSNARE, rSec22b, which is not present in the intestinal PCTV [71,76,77]. Gusarova et al. also have reported that the exit of apoB100 from ER membranes in the rat liver requires COPII proteins but that the size of the lipoprotein entering the vesicle is small with further lipidation occurring downstream from COPII dependent events [78]. Interestingly, patients with CLSD (SEC23A mutation) are not reported to have decreased cholesterol levels, malabsorption or hepatic steatosis which could point to the importance of the different COPII coat paralogues in the PTV, PCTV and VTV transport systems. Differences in the protein composition of the PTV, PCTV and VTV vesicles could also account for the differences in their cargo proteins. Taken together, the data from AD/CMRD patients suggest that specific sequences of SAR1B are essential for the intracellular processing of intestinal chylomicrons and are less important for hepatic VLDL secretion.

A final remark concerns the amount and the localization of the Sar1 protein observed by immunostaining. Sar1 immunostaining was found to localize heterogeneously in the *villi *and in the enterocytes of both healthy individuals and patients. Immunostaining was frequently observed on only one side of the villus and was concentrated in the apical region of the enterocytes. This pattern has been described previously for lipid droplets in AD, in CRD- Marinesco-Sjoren syndrome [14,15] as well as for proteins involved in the chylomicron secretory pathway, including hepatic FABP [79] which is purported to be involved in the budding step of the PCTV [32]. This pattern is reminiscent of the mosaicism observed for other proteins [80-82]. Whether this distribution is related to several populations of enterocytes arising from different stem cells [83] or to some other physiological mechanism is not known. Further, although the I and L-FABP genes have been reported as not being responsible for AD [14], L-FABP has been shown to be present but in reduced amounts in the enterocytes of AD patients as assessed by immunohistochemical analysis [79]. The reason for this and its possible role in the pathology of AD remains unexplained.

The complexity of the secretory machinery and the numerous combinations of the different paralogues suggests that phenotypes similar to AD/CMRD could arise from other mutations in either tissue specific or common elements belonging to the intestinal and hepatic apoB-containing lipoprotein secretory pathways. We have, in fact, now found four families with 6 affected individuals that exhibit the AD/CMRD phenotype in which the *SAR1B *gene is normal (unpublished results). We are currently in the process of defining the genetic defect in these families.

## Competing interests

The authors declare that they have no competing interests.

## Authors' contributions

Clinical care of the patients, generation and analysis of the clinical data, AG, AM and MES. Patient recruitment AM and TA. Data handling and management: intestinal expression of SAR1A and SARA1B by QPCR, JB, LPA. Lipid and lipoprotein analysis, DB, EB. Intestinal histology and immunochemistry, JC. Ultrastructural analysis, JCG and MES. PCSK9, SAR1A, SAR1B gene sequencing JPR, MA, MV, CB. Critical revision of the paper, CB. Concept and design of the study: supervising the project and writing of the manuscript, LPA and MES. All authors participated in the context of their individual expertise to the writing of the paper, read and approved the final version of the article.

## Supplementary Material

Additional file 1 **List of mutations in the *SAR1B *gene reported in AD/CMRD patients**. List of individuals for whom a mutation in the *SAR1B *gene has been described along with the predicted amino acid change, predicted effect on the protein and references. Click here for file

Additional file 2 **SAR1B structural and functional domains**. The sequence and secondary structure of human SAR1B and the functional roles that have been attributed to regions of the protein (based upon the data in the references cited) are shown along with the positions of the mutations identified in cases of Anderson's Disease/Chylomicron Retention Disease. Click here for file

## Aknowledgements

The authors thank Pr J Schmitz (Département de Pédiatrie, Hôpital Necker-Enfants Malades, AP-HP, 75015 Paris) for invaluable brain storming in order to understand these 3 new cases of Anderson's disease, MC Federspiel (UF de Biochimie des Maladies Métaboliques, Service de Biochimie Métabolique, Groupe Hospitalier Pitié-Salpêtrière, AP-HP), S. Faïna (Service de Biochimie et Génétique Moléculaire, CHU A Paré, AP-HP et Faculté de Médecine (PIFO-UVSQ), Boulogne) for their expert technical assistance in lipoprotein analysis and *PCSK9 *gene sequencing respectively, D Thiébaut (Laboratoire d'Anatomie et Cytologie Pathologiques CHU Brabois, Nancy) for her technical assistance in immunochemistry and electron microscopy and JP Laigneau (INSERM, Faculté de Médecine X Bichat, Paris, France) for his expertise in illustration.

Financial support

These studies were supported by INSERM (AG, JB, JPR, MA, CB, AM, MV, MES), CNRS (LPA) and by grants from PHRC (AOMO6024), (JB, JPR, MA, CB, MV, MES) and ANR (ANR-08-GENO-002-01), (JB, JPR, MA, CB, MV, MES).
