# Supplementary material for: Molecular analysis and intestinal expression of SAR1 genes and proteins in Anderson's disease (Chylomicron retention disease)
Source: Orphanet J Rare Dis. 2011 Jan 14;6:1. doi: 10.1186/1750-1172-6-1 (PMC3029219; doi:10.1186/1750-1172-6-1)
Supplement: Additional file 1 — List of mutations in the SAR1B gene reported in AD/CMRD patients. List of individuals for whom a mutation in the SAR1B gene has been described along with the predicted amino acid change, predicted effect on the protein and references. [file 1750-1172-6-1-S1.DOC]

# Additional file 1: List of mutations in the *SAR1B* gene reported in AD/CMRD patients

| Family Person | Diagnosis | Origin | Sex | Status | Mutation | Exon* | Predicted amino acid change | Predicted effect on protein | References |
| --- | --- | --- | --- | --- | --- | --- | --- | --- | --- |
| 1-1 | AD | Algerian | M | Hom. | c.109G>A | 4 | p.Gly37Arg | No affinity for GDP/GTP. Mutation in a conserved guanine nucleotide binding motif. | 14,18 |
| 1-2 | AD | Algerian | F | Hom. |  |  |  |  |  |
| 2-1 | CMRD | Canadian | M | Hom. | c.409G>A | 7 | p.Asp137Asn | Reduced affinity for GDP/GTP. Mutation in a conserved guanine nucleotide binding motif. | 8, 18 |
| 2-2 | CMRD | Canadian | F | Hom. |  |  |  |  |  |
| 3-1 | CMRD | Canadian | M | Het. | c.409G>A | 7 | p. Asp137Asn | Reduced affinity for GDP/GTP. Same as 2-2. | 8, 18 |
|  |  |  |  | Het. | c.83_84delTG | 4 | p.Leu28fsX7 | Translation arrested after 34 residues. Loss of most functions. |  |
| 4-1 | CMRD | Canadian | F | Hom. | c.537T>A | 8 | p.Ser179Arg | No affinity for GDP/GTP. Affects H-bonds in GTP binding site. | 8, 18 |
| 5-1 | CMRD | Turkish | M | Hom. | c.555_558dupTTAC | 8 | p.Gly187fsX13 | Change in the amino acids forming helix 6 and reduced affinity for the ER membrane. The mutated allele replaces amino acids 187-198 of SAR1B with the amino acid sequence LRRRLPLDGTVH. Affects C-terminal residues that cover the hydrophobic core of the central beta sheet and probably affects the structural integrity of the protein. | 12,18 |
| 5-2 | CMRD | Turkish | F | Hom. |  |  |  |  |  |
| Family  Person | Diagnosis | Origin | Sex | Status | Mutation | Exon* | Predicted amino acid change | Predicted effect on protein | References |
| 6-1 | CMRD-MSS | Italian | M | Hom. | c.349-1G>C (A homozygous 4-nucleotide duplication, 506_509dupAAGA, in exon 6 of the SIL1 gene is found here and in all MSS patients) | 7 | Null allele, p.Asp116VSX119, p.Ser117_Lys160del | The outcome of this mutation could include exon skipping, activation of a nearby cryptic splice site or production of an unspliced mRNA. No translated product. Translation arrested after 118 residues. Loss of secondary structure. | 15,18, 20 |
| 6-2 | CMRD-MSS | Italian | M | Hom. |  |  |  |  |  |
| 7-1 | CMRD- AD | Pakistan | F | Hom. | c.[536G>T; 542T>C] | 8 | p.Ser179Ile p.Leu181Pro | No affinity for GDP/GTP. Affects H-bonds in GTP binding site and hydrophobic packing between Leu181 and purine ring of guanine base. Each parent is heterozygous on one allele with respect to both sequence | 18 |
| 8-1 | AD | Moroccan | M | Hom. | c.109G>A | 4 | p.Gly37Arg | No affinity for GDP/GTP. Same as 1-1. | 6, 18 |
| 9-1 | CMRD- AD | Algerian | M | Hom. | c.1-4482_58+1406del5946 ins15bp c.1-4482_58+1406del5946 insCGCATGATGGCGGGT | 3 | p.Met1_His43del | Deletion exon 3, protein lacks the first 43 amino acids important for membrane interaction and Sec12 interaction. | 19 |
| 9-2 | CMRD- AD | Algerian | F | Hom. |  |  |  |  |  |

| Family  Person | Diagnosis | Origin | Sex | Status | Mutation | Exon* | Predicted amino acid change | Predicted effect on protein | References |
| --- | --- | --- | --- | --- | --- | --- | --- | --- | --- |
| 10-1 | CMRD- AD | Turkish | M | Hom. | c.364G>C | 7 | p.Glu122X | Protein predicted to have 122 residues, lacks the last 76 amino acids. Affects interaction with membrane and control of membrane constriction. | 19 |
| 10-2 | CMRD- AD | Turkish | F | Hom. |  |  |  |  |  |
| 10-3 | CMRD- AD | Turkish | F | Hom. |  |  |  |  |  |
| 10-4 | CMRD- AD | Turkish | M | Hom. |  |  |  |  |  |
| 11-1 | CMRD-  AD | French-Canadian | M | Hom. | c.409G>A | 7 | p.Asp137Asn | Mutation in the recognition site for guanine base essential for GTPase activity. Same as 2-1. | 19 |
| 11-2 | CMRD- AD | French-Canadian | M | Hom. |  |  |  |  |  |
| 12-1 | CMRD- AD | French-Canadian | F | Hom. |  |  |  |  |  |
| Family  Person | Diagnosis | Origin | Sex | Status | Mutation | Exon* | Predicted amino acid change | Predicted effect on protein | References |
| 13-1 | CMRD- AD | French-Canadian | M | Het. | c.409G>A | 7 | p.Asp137Asn | Mutation in the recognition site for guanine base essential for GTPase  activity. Same as 2-1. | 19 |
|  |  |  |  | Het. | c.537T>A | 8 | p.Ser179Arg | No affinity for GDP/GTP. Affects H-bonds in GTP binding site. Same as 4-1. |  |
| 13-2 | CMRD- AD | French-Canadian | F | Het. | c.409G>A | 7 | p.Asp137Asn | Mutation in the recognition site for guanine base essential for GTPase activity. Same as 2-1. | 19 |
|  |  |  |  | Het. | c.537T>A | 8 | p.Ser179Arg | No affinity for GDP/GTP. Affects H-bonds in GTP binding site. Same as 4-1. |  |
| 14-1 | CMRD- AD | French-Canadian | F | Hom. | c.537T>A | 8 | p.Ser179Arg | No affinity for GDP/GTP. Affects H-bonds in GTP binding site. Same as 4-1. | 19 |
| 14-2 | CMRD- AD | French-Canadian | M | Hom. |  |  |  |  |  |
| 15-1 | CMRD- AD | French-Canadian | F | Hom. |  |  |  |  |  |
| Family  Person | Diagnosis | Origin | Sex | Status | Mutation | Exon* | Predicted amino acid change | Predicted effect on protein | References |
| 16-1 | CMRD- AD | Portuguese | F | Hom. | c.554G>T | 8 | p.Gly185Val | Near alpha helix 6 in region that juxtaposes the ER membrane. | 19 |
| 17-1 | AD | French | F | Hom. | c.499G>T | 8 | p.Glu166X | Protein predicted to have 166 residues, lacks the last 32 residues and a region that juxtaposes the ER membrane. | 21 |
| 17-2 | AD | French | F | Hom. |  |  |  |  |  |
| 18-1 | CMRD | Moroccan | F | Hom. | c.83_84delTG | 4 | p.Leu28fsX7 | Protein predicted to have only 34 residues and would lack most of the functional regions of the protein. Same as 3-1. | 23 |
| 18-2** | Asymptomatic** | Moroccan | F | Hom. |  |  |  |  |  |
| 19-1 | CMRD | ? Thailand | M | Het. | c.32G>A | 3 | p.Gly11Asp | Mutation in the N-terminal Sec-12 interacting site (residues 1-19). | 22 |
|  |  |  |  | Het. | c.224A>G | 5 | p.Asp75Gly | Mutation in the GTP hydrolysis active site (residues 75-78). |  |
| 20-1 | AD- PCSK9 | Moroccan | F | Hom. | c.83_84delTG (The patient is also a heterozygous carrier of a 3 base duplication, c.61_63dupCTG, in exon 1 of the PCSK9 gene) | 4 | p.Leu28ArgfsX7 | Protein predicted to have only 34 residues and would lack most of the functional regions of the protein. Same as 3-1.. | Current study |
| Family  Person | Diagnosis | Origin | Sex | Status | Mutation | Exon* | Predicted amino acid change | Predicted effect on protein | References |
| 21-1 | AD | Turkish | M | Hom. | c.142delG | 4 | p.Asp48ThrfsX17 | Protein predicted to have only 64 residues and would lack many of the functional regions of the protein. | Current study |
| 21-2 | AD | Turkish | F | Hom. |  |  |  |  |  |
| *The exon numbering has been updated to be consistent with the information available as of May 2010. | | | | | | | | | |
| **18-2 is the mother of 18-1 and is homozygous but asymptomatic. | | | | | | | | | |
