# Supplementary material for: Molecular analysis and intestinal expression of SAR1 genes and proteins in Anderson's disease (Chylomicron retention disease)
Source: Orphanet J Rare Dis. 2011 Jan 14;6:1. doi: 10.1186/1750-1172-6-1 (PMC3029219; doi:10.1186/1750-1172-6-1)
Supplement: Additional file 2 — SAR1B structural and functional domains. The sequence and secondary structure of human SAR1B and the functional roles that have been attributed to regions of the protein (based upon the data in the references cited) are shown along with the positions of the mutations identified in cases of Anderson's Disease/Chylomicron Retention Disease. [file 1750-1172-6-1-S2.DOC]

**Additional file 2: SAR1B structural and functional domains**

**Modified C-terminal**

**Truncated proteins**

**10 20 30 40 50 60 70 80 90 100 110 120 130 140 150 160 170 180 190**

**MSFIFDWIYS GFSSVLQFLG LYKKTGKLVF LGLDNAGKTT LLHMLKDDRL GQHVPTLHPT SEELTIAGMT FTTFDLGGHV QARRVWKNYL PAINGIVFLV DCADHERLLE SKEELDSLMT DETIANVPIL ILGNKIDRPE AISEERLREM FGLYGQTTGK GSISLKELNA RPLEVFMCSV LKRQGYGEGF RWMAQYID**

**Location of mutations in Anderson’s Disease/Chylomicron Retention Disease**

**STAR motif : Hydrophobic patch which facilitates the stable recruitment of both Sar1 and Sec23(GAP)/24 to the membrane and the recognition of mSec12.**

**Switch 1 region: Surface loop that is predicted to undergo conformational change upon GTP binding. Mg**^2+^ **is coordinated by the γ phosphate of GTP and the hydroxyl of Thr56.**

**Switch 2 region: Important for the hydrolysis of GTP. Mg**^2+^ **is coordinated by the γ phosphate of GDP and interacts with the backbone of Gly76. Mutation of His79 inhibits the hydrolysis of GTP and the disassembly of the COPII complex.**

**Ω loop: Acts in the control of membrane constriction.**

**α1’helix: Affects Sec23 promoted hydrolysis, essential to anchor Sar1 to the membrane, proposed to interact with Sec12 and may be involved in membrane deformation and vesicle fusion.**

**Guanine nucleotide binding motifs: Mg**^2+^ **is coordinated by the β phosphate of GDP and the hydroxyl of Thr39. Mutation of Thr39 to Asn results in reduced affinity for GTP, and interferes with the interaction with Sec12 (GEF). Mutation of Asp34 in yeast interferes with GTP loading and destroys the ability of Sar1 to function in vesicle budding. Mutation of Asn134 to Ile also inhibits ER to Golgi transport.**

**Regions of Sar1 that interact with Sec23.**

**Residues important for initial events of COPII vesicle budding related to Sar1 recruitment and/or activation.**

**Regions of Sar1 that juxtapose the ER membrane.**

**Regions of Sar1 that interact with Sec31.**

**Alpha helical structure.**

**Beta sheet structure.**

**References: 1-28,75; K.R. Long,Y. Yamamoto, A.L. Baker, S.C. Watkins, C.B. Coyne, J.F. Conway, M. Aridor: Sar1 assembly regulates membrane constriction and ER export. J Cell Biol (2010) 190:115-128; X. Bi, R.A. Corpina, J. Goldberg: Structure of the Sec23/24-Sar1 pre-budding complex of the COPII vesicle coat. Nature (2002) 419: 271-277; C. Russell, S.M. Stagg: New insights into the structural mechanisms of the COPII coat. Traffic (2010) 11:303-310; X. Bi, J.D. Mancias, J. Goldberg: Insights into COPII coat nucleation from the structure of Sec23*Sar1 complexed with the active fragment of Sec31. Dev Cell (2007) 13:635-645; M. Huang, J.T. Weissman, S. Béraud-Dufoour, P. Luan, C. Wang, W. Chei, M. Aridor, I.A. Wilson, W.E. Balch: Crystal structure of Sar1-GDP at 1.7 A resolution and the role of the NH2 terminus in ER export. J Cell Biol (2001) 6: 937-948; Y. Rao, C. Bian, C. Yuan, Y. Li, L. Chen, X. Ye, Z. Huang, M. Huang: An open conformation of switch I revealed by Sar1-GDP crystal structure at low Mg2+. Biochem Biophys Res Comm (2006) 348:908-915; M. Aridor, K.N. Fish, S. Bannykh, J. Weissman, T.H. Roberts, J. Lippincott-Schwartz, W.E. Balch: The Sar1 GTPase coordinates biosynthetic cargo selection with endoplasmic reticulum export sort site assembly. J Cell Biol (2001) 152: 213-229; M. Hanzal-Bayer, L. Renault, P. Roversi, A. Wittinghofer, R.C. Hillig:** [**The complex of Arl2-GTP and PDE delta: from structure to function.**](http://www.ncbi.nlm.nih.gov.gate1.inist.fr/pubmed/11980706) **EMBO J (2002) 21: 2095-106; W. Kremer, G. Steiner, S. Béraud-Dufour, H.R. Kalbitzer:** [**Conformational states of the small G protein Arf-1 in complex with the guanine nucleotide exchange factor ARNO-Sec7.**](http://www.ncbi.nlm.nih.gov.gate1.inist.fr/pubmed/14739276) **J Biol Chem (2004) 279:17004-12; S. Pasqualato, L. Renault, J Cherfils:** [**Arf, Arl, Arp and Sar proteins: a family of GTP-binding proteins with a structural device for 'front-back' communication.**](http://www.ncbi.nlm.nih.gov.gate1.inist.fr/pubmed/12429613) **EMBO Rep (2002)11:1035-41; K. Wennerberg, K.L. Rossman, C.J. Der: The Ras superfamily at a glance. J Cell Science (2005) 118: 843-846; J. Colicelli: Human RAS superfamily proteins and related GTPases. SciSTKE (2004) 250: RE13; S. Pandit, N. Srinivasan: Survey for G-proteins in the prokaryotic genomes: prediction of functional roles based on classification. Proteins (2003) 52: 585-597; J. Bonifacino, B.S. Glick: The mechanisms of vesicle budding and fusion. Cell (2004) 116: 153-166.**
